# Herpes B virus gD interaction with its human receptor – an *in silico* analysis approach

**DOI:** 10.1186/1742-4682-11-27

**Published:** 2014-06-06

**Authors:** Lingke Li, Zhengliang Qiu, Yan Li, Feng Liang, Huahu Ye, Yongqin Cai, Wanfeng Guo, Yan Li, Junjie Yue

**Affiliations:** 1 Hospital No.307 of PLA, the Academy of Military Medical Sciences, Beijing 100071, China; 2 Department of Cardiovascular Surgery, Air force General Hospital, Beijing 100142, China; 3 Laboratory Animal Center of the Academy of Military Medical Science, Beijing 100071, China; 4 Hospital No.309 of PLA, Beijing 100091, China; 5 Beijing Institute of Biotechnology, Beijing 100071, China

**Keywords:** Herpes B virus, Glycoprotein D, Receptor, Interaction, Complex

## Abstract

**Background:**

The glycoprotein D (gD) is essential for Herpes B virus (BV) entry into mammalian cells. Nectin-1, an HSV-1 gD receptor, is found to be the receptor which mediated BV induced cell-cell fusion, while HVEM does not mediate fusion by BV glycoprotein. However, the specific sequence and structural requirements of the BV gD for the recognition of and binding to Nectin-1 are unknown. Moreover, the 3D structures of BV gD and the BV gD-receptor complex have not been determined. In this study, we propose a reliable model of the interaction of the BV gD with receptor using bioinformatics tools.

**Results:**

The three-dimensional structures of two BV gD-receptor complexes were constructed using homology modelling and docking strategy. Based on the models of these complexes, the BV gD receptor interaction patterns were calculated. The results showed that the interface between the BV gD and nectin-1 molecule is not geometrically complementary. The computed molecular interactions indicated that two terminal extensions were the main region of BV gD that binds to nectin-1 and that hydrophobic contacts between the two molecules play key roles in their recognition and binding. The constructed BV gD-HVEM complex model showed that this complex had a lower shape complementarity value and a smaller interface area compared with the HSV-1 gD-HVEM complex, and the number of intermolecular interactions between BV gD-HVEM were fewer than that of HSV-1 gD-HVEM complex. These results could explain why HVEM does not function as a receptor for BV gD.

**Conclusion:**

In this study, we present structural model for the BV gD in a complex with its receptor. Some features predicted by this model can explain previously reported experimental data. This complex model may lead to a better understanding of the function of BV gD and its interaction with receptor and will improve our understanding of the activation of the BV fusion and entry process.

## Background

Herpes B virus (BV) is a member of genus Simplexvirus within the Alphaherpesvirinae subfamily. It has also been known as Cercopithecine herpes virus 1, monkey B virus, B virus or Herpes B Monkey virus [1]. BV naturally infects macaque monkeys and generally causes only mild localized or asymptomatic infections [2]. When transmitted to foreign hosts, such as humans or monkey species other than macaques, B virus often results in severe pathogenesis, including paralysis, encephalitis, encephalomyelitis, and death [3-5]. Historically, BV has a fatality rate greater than 70% in untreated human BV infection cases [6].

Because of the high mortality of its infection in untreated humans and the lack of vaccines, BV is classified as a biosafety level 4 (BSL-4) pathogen [7,8]. Owing to the biosafety concerns, little research has been conducted directly using BV; most of the knowledge on this virus is extrapolated from study of its closely related viral species of the subfamily Alphaherpesvirinae, herpes simplex virus (HSV) type 1 (HSV-1) and HSV-2 [6].

BV has been shown to display many similar biological characteristics with HSV-1 and HSV-2 [9-11]. The genomes of BV and HSV-1 and HSV-2 are made up by homologous genes in the same order and orientation [2,12,13]. The similarity is at an average of approximately 62% identity of amino acid residues for all homologous proteins of herpes B shares with HSV-1 and HSV-2. In its natural host, B virus exhibits similar signs of infection to that observed on HSV infections in humans. Similarly to the high pathogenicity of BV in humans, HSV infection of marmosets can also be fatal [14].

HSV entry into target cells occurs primarily through the interactions of viral envelope glycoproteins with a variety of cellular receptors. There are 11 known enveloped proteins on the virus surface of HSV-1 and a minimum four of them are believed to be involved in HSV entry and fusion. Among them, glycoprotein D (gD) is believed to have an important role for virus entry into mammalian cells.

It is widely accepted that binding of HSV-1 gD to a cell surface receptor triggers the conformational changes in other viral glycoproteins leading to membrane fusion and viral entry [15,16]. Up to now, at least four HSV receptors have been identified: herpesvirus entry mediator (HVEM), nectin-1, nectin-2, and 3-O-sulfated heparan sulfate [17-20].

A previous study shows that nectin-1 mediates fusion of cells expressing glycoprotein from BV while HVEM does not mediate fusion by BV glycoprotein [21]. In other words, nectin-1 is the “master” receptor for B virus infection [22].

To further establish the molecular basis of BV fusion to the host cell, a structural view of the interaction between gD and nectin-1 should be obtained. Although no structural information on BV gD has not been reported, the previously reported structures of HSV-1 gD alone and bound to nectin-1 provide an opportunity to propose a structural model for BV gD-receptor complex.

The aim of this study is to define a structural model for the BV gD-nectin-1 complex and to describe the interaction between BV gD and nectin-1 using computational methods. Therefore, we can identify and characterise the binding region within BV gD that binds to its surface receptor and understand required interactions between the glycoprotein and its receptor critical for virus fusion and entry.

## Results

### Homology modeling of BV gD

**Figure 1:**
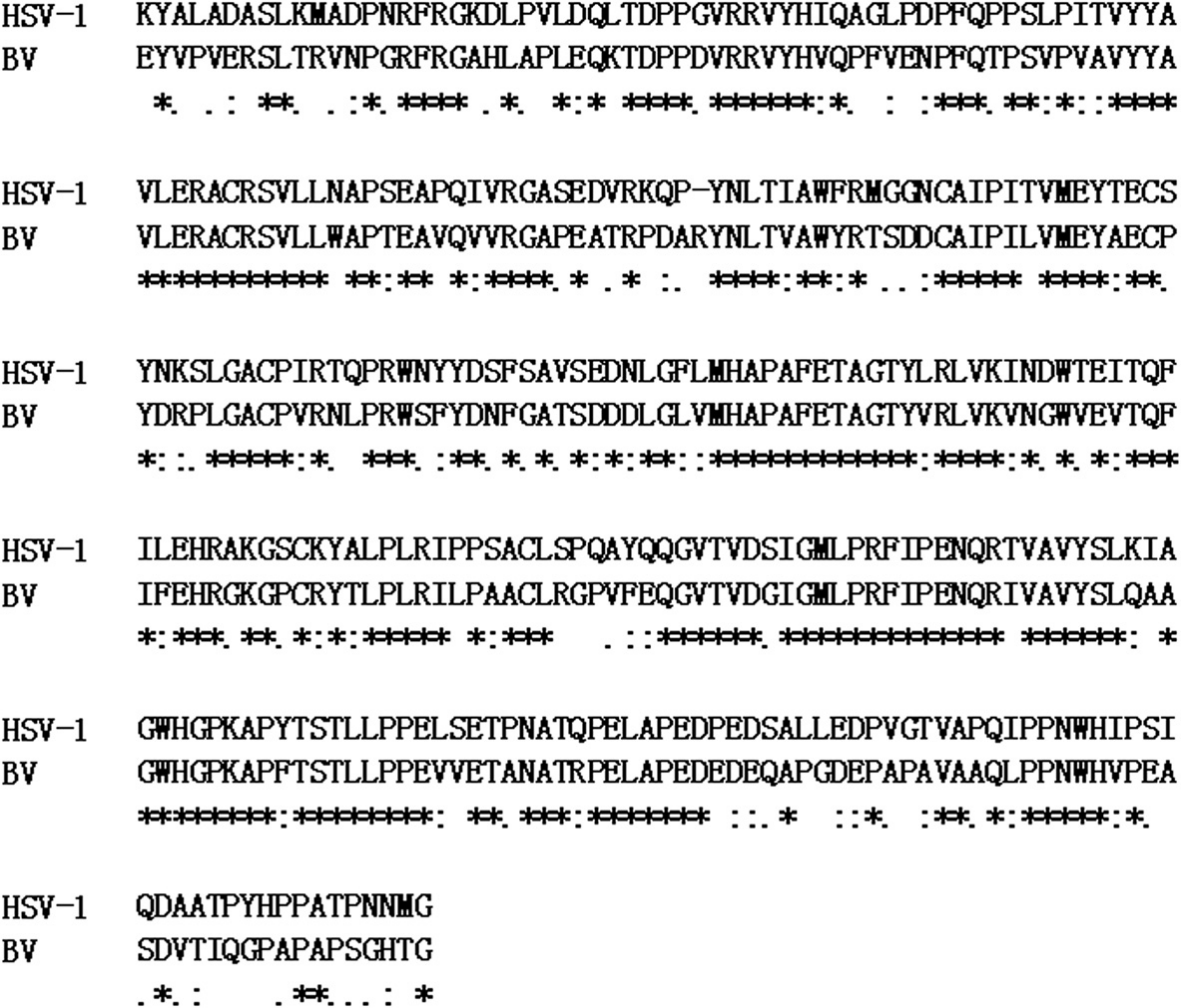
**Sequence alignment result of BV gD with the template HSV-1 gD.** “*” represents identical amino acids, “:” represents strong similarity, “.” represents weaker similarity, and blank represents different amino acids or gap.

**Figure 2:**
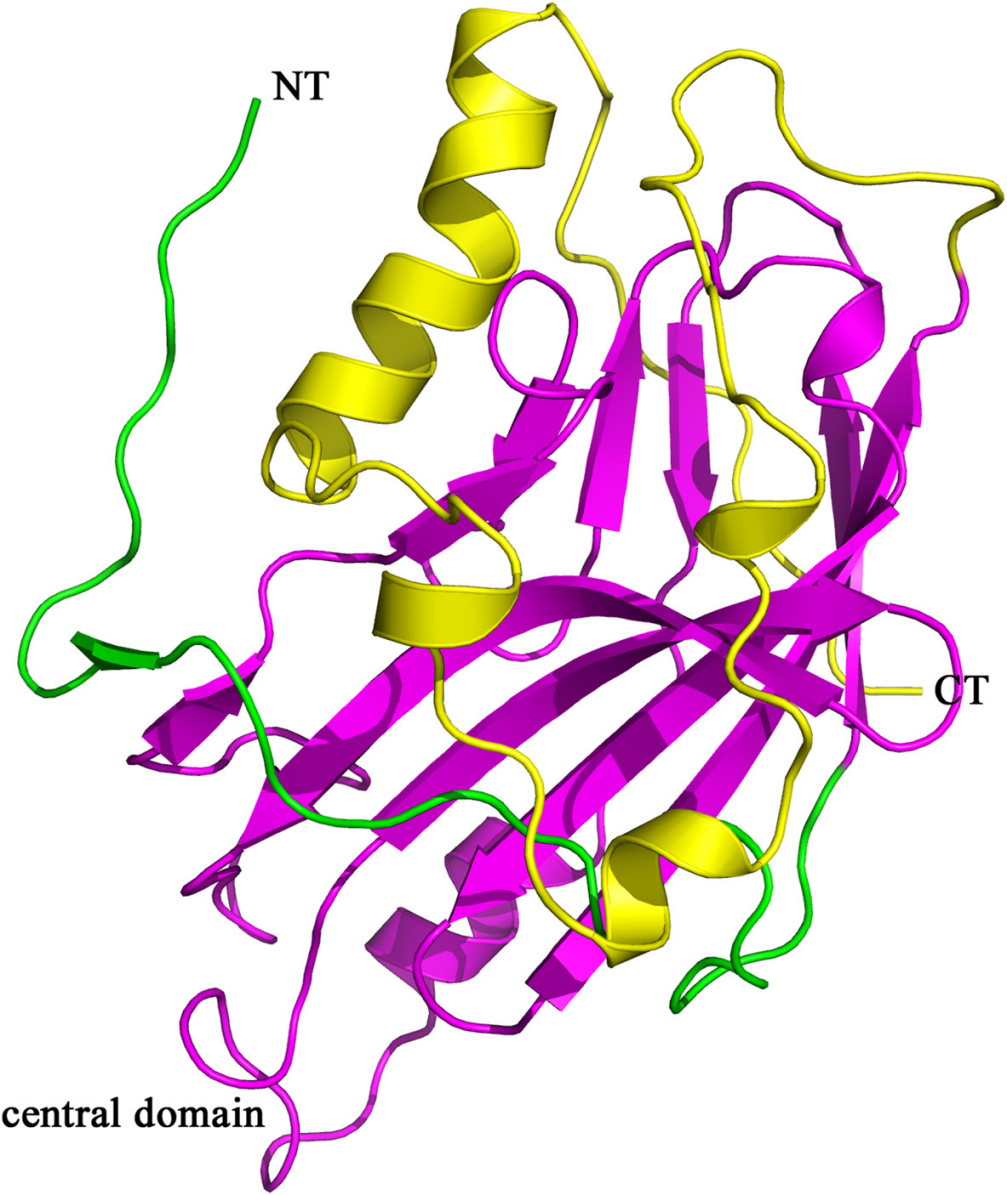
Ramachandran plot of the structure of BV gD.

Up to now, there is no structural information on either BV gD or the BV gD-receptor complex. The purpose of this study is to model the structural features of BV gD that interact with its receptor. As the first step, the three dimensional structure of BV gD should be modeled. The template identification was performed by a BLAST search against PDB. Among the BLAST hits, its ortholog in HSV-1 shows the highest similarity with the amino acid sequence of BV gD. The structures of HSV-1 gD both in apo and receptor bound form had been solved [15,23,24]. The sequence identities between HSV-1 gD and BV gD are up to 62%. The sequence alignment of HSV-1 gD and BV gD is shown in Figure 1. The asterisk represents identical amino acids present in two protein sequences.In total, 3D model of BV gD was generated using the structure of HSV-1 gD with MODELER. The templates selected for homology modeling was the HSV-1 gD crystal structure in nectin-1 binding form (PDB code 3U82). The structure of BV gD was similar to that of HSV-1 gD, with an r.m.s. deviation of 2.34 Å for 231 Cα positions (residues 24–254). In the predicted BV gD structure (Figure 2), the central Ig-like V domain (residues 57–185) is comprised of nine strands, and flanked by the N- and C-terminal extensions. The N-terminal extension (residues 24 to 56) of gD forms a hairpin structure. The C-terminal extension (residues 186 to 254) includes four helices and few loops. The two terminal extensions constitute a big, flat surface.

**Figure 3:**
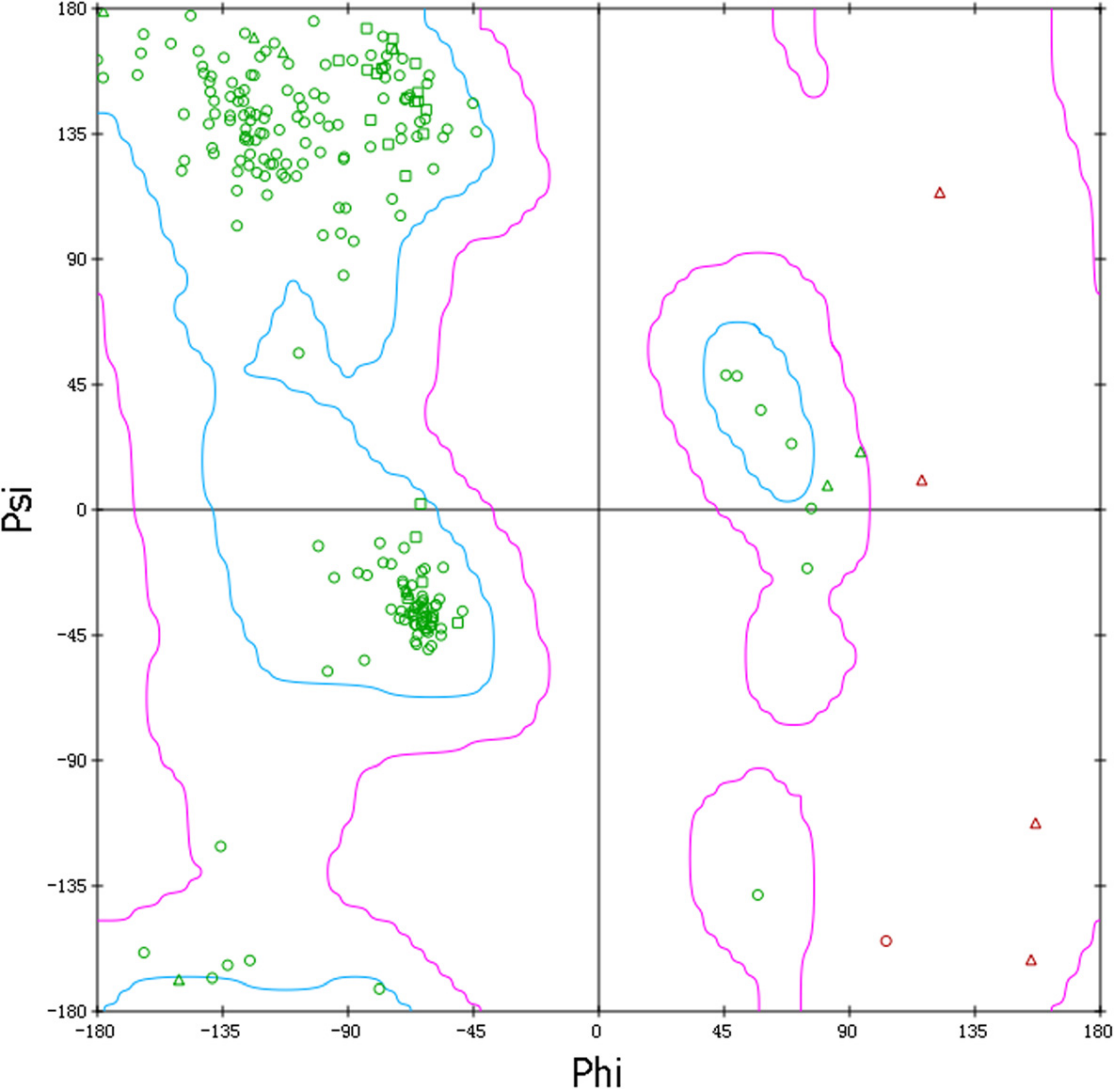
**The BV gD structure obtained by homology modelling method.** The structure of BV gD is shown in carton; The N-terminal extension (residues 24 to 56) is colored green, the C-terminal extension (residues 186 to 254) is colored yellow, and the central Ig-like V domain is colored magenta.

The quality of the predicted BV gD structure was evaluated using the Profiles-3D [25] and Ramachandran Plot method [26] in Discovery Studio 2.5 (Accelrys Inc.). The two protocols validate a protein model by performing full geometric analysis as well as stereo chemical quality of a protein structure. The Profiles-3D checks the validity of a protein structure by measuring the compatibility of a 3D structure with the sequence of the protein. This is a useful measure of the overall quality of the structure. The Ramachandran plot provides an easy way to view the distribution of torsion angles of a protein structure. The two torsion angles of a polypeptide chain describe the rotations of the polypeptide backbone around the bonds between N-Cα and Cα-C, which are among the most important local structural parameters that control protein folding. The Ramachandran plot serves as an important indicator of the quality of protein three-dimensional structures by providing an overview of allowed and disallowed regions of torsion angle values.According to the result from Ramachandran Plot, 91.2% residues of the predicted BV gD model were located in the favored region, 8.3% in the additional allowed region, 0.5% in the generously allowed region and no non-Gly residues were found in the disallowed region (Figure 3). The Profiles-3D program gives the similar result. The Profiles-3D Score of this predicted BV gD model is 92.0, which is higher than the Verify Expected Low Score (47.2). This suggests that overall quality of the model as a whole is largely correct.

Therefore, the Profiles-3D and Ramachandran Plot results indicate that the predicted BV gD model can be considered of good quality and employed for further analyses.

### The binding region of BV gD to its human receptor

Herpes B virus entry into target cells requires virus encoded glycoprotein interact with cellular receptors to facilitate virus entry. A previous study reported that Herpes B Virus only utilized human nectin-1 but not HVEM for cell-cell fusion and virus entry by BV glycoprotein D [21,22]. To identify and characterize the binding region within BV gD that binds to nectin-1 and further determine which specific residues are critical for receptor binding, we constructed the BV gD-nectin-1 complex model using computational methods.

The predicted BV gD-nectin-1 complex has a shape complementarity (SC) value of 0.53, which is slightly higher than the value seen in the HSV-1 gD-nectin-1 complex (0.44). These values are lower than those typically seen in protein receptor-ligand complexes, which have the common shape complementarity values within the range of 0.62 to 0.69 [27]. This indicates that the interfaces between nectin-1 and the two gD molecules are of low degree of geometrically complementarity.

The interface area of the modeled BV gD-nectin-1 complex had a value about 1646 Å^2^, suggesting that the complex had the “standard-size” interface observed in other protein/protein complexes and viral glycoprotein/receptor complexes [24,28-31]. The interface area contributed by the non-polar atom was 984 Å^2^, which was 59.8% of the total interface area, which hints that the hydrophobic contacts between BV gD and nectin-1 play key roles in their recognition and binding.

A detailed analysis of the interactions between the BV gD and nectin-1 shows their binding sites. The residues involved in the protein-protein interface of the BV gD-nectin-1 complex were determined on the basis of the buried surface area. There were about 25 residues in gD and 29 residues in nectin-1 that lost their accessible surface in the complex. In the complex structure, the BV gD forms extensive interactions with nectin-1, including 4 hydrogen bonds (Table 1), 1 salt bridge (Table 2), and 48 hydrophobic contacts (Table 3).

**Table 1:** Hydrogen bond interactions observed in the complex between BV gD- human nectin-1 complex

**Donor atom**	**Acceptor atom**	**Distance**	**Angle XDA**	**Angle DAY**
NECTIN-1:Glu64:NE2	BV gD:Pro222:O	2.93	92.97	123.59
BV gD:Arg223:NH2	NECTIN-1:Glu125:OE1	2.75	113.59	129.92
NECTIN-1:Asn77:ND2	BV gD:Asp216:O	2.56	100.66	114.01
NECTIN-1:Asn77:N	BV gD:Asp216:O	2.55	100.17	157.59

**Table 2:** Salt bridge observed in the complex between BV gD- human nectin-1 complex

**BV gD residue**	**Nectin-1 residue**
Arg223	Glu125

**Table 3:** Hydrophobic interactions observed in the complex between BV gD- human nectin-1 complex

**BV gD residues**	**Human nectin-1 residues**
Pro24(2)	Pro130(2)
Gln27(1)	Phe129(1)
Arg36(3)	Met85(3)
Val37(2)	Met85(2)
Tyr38(4)	Ile80(1),Asn82(1), Met85(2)
His39(1)	Leu90(1)
Leu199(2)	Gly73(2)
Asp216(14)	Asn77(5),Leu90(9)
Gly217(2)	Gln76(2)
Gly219(2)	Asn77(2)
Leu221(2)	Thr66(2)
Pro222(2)	Gln64(2)
Phe224(4)	Thr63(4)
Ile231(1)	Phe129(1)
Val232(2)	Phe129(2)
Tyr234(4)	Pro130(4)

There are a total of 48 hydrophobic contacts. Numbers in parentheses refer to the number of hydrophobic contacts.

**Figure 4:**
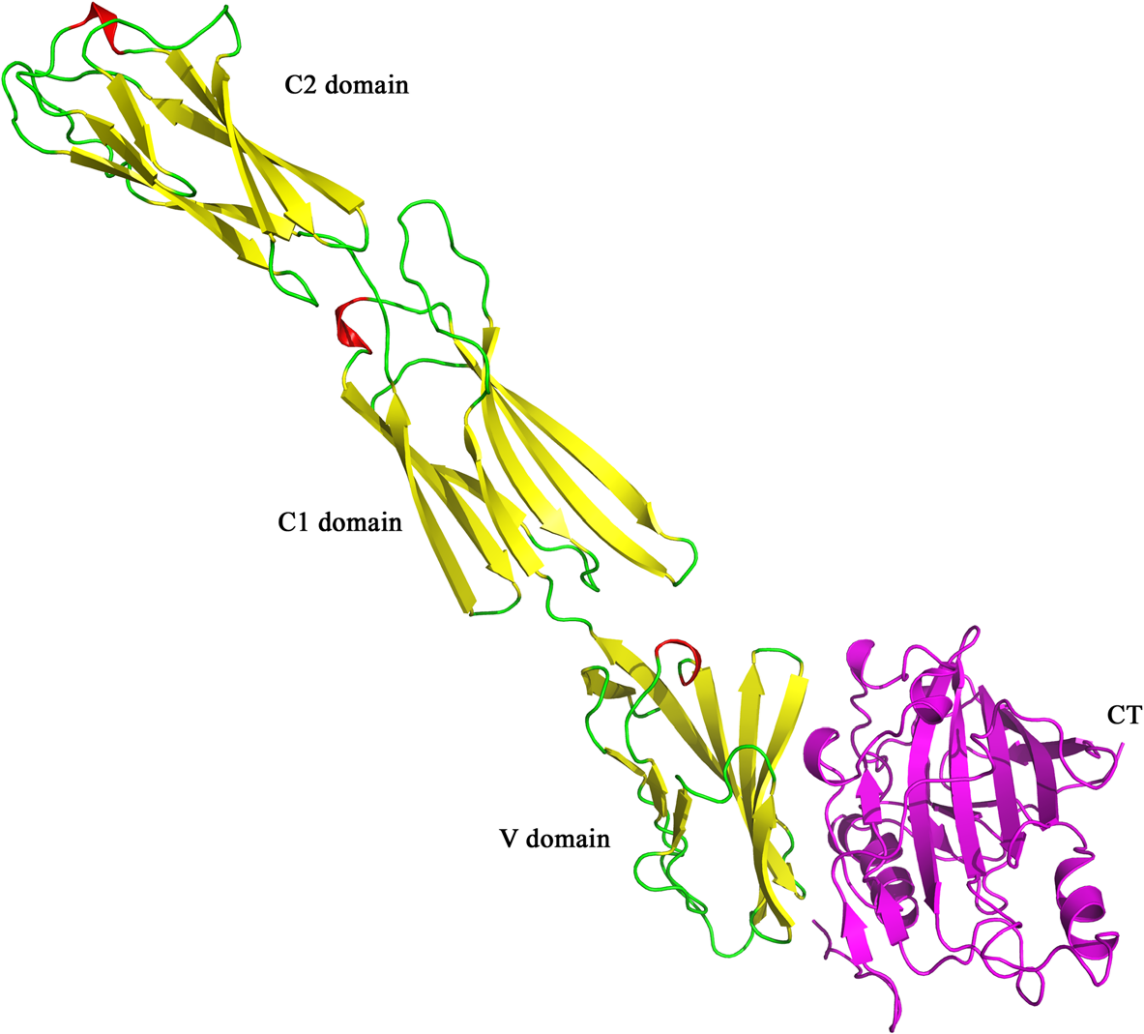
**The structure of BV gD-human nectin-1 complex.** Both the BV gD and human nectin-1 are shown in carton. The helical residues of nectin-1 are colored red, and beta sheet residues are coloured yellow, and the loop and unassigned residues are coloured green. BV gD is coloured magenta.

The BV gD contributes 764.8 Å^2^ of the buried surface area, and makes less contribution on the interacting interface than the nectin-1 in their binding. The receptor binding sites of BV gD consists of 4 discontinuous segments, including residues 24–27, 35–39 of the N-terminal extension and residues 216–224 and 231–235 the C-terminal extension (Figure 4). The four loops form a large, flat surface to accommodate interacting residues of nectin-1. Among the 25 residues of BV gD, residues 38Tyr, 216Asp, 223Arg, 224Phe, were important for nectin-1 interaction, and they established more direct and closer contact with the CpG ODN molecule. Residues 38Tyr, 235Tyr and 224Phe form four hydrophobic interactions with nectin-1, respectively. Residues 216Asp forms two hydrogen bonds with Asn78 in nectin-1.In particular, 223Arg forms a salt bridge with and a hydrogen bond with Glu126 in nectin-1. The salt bridge between 223Arg and residue Glu126 in nectin-1 was also observed in HSV-1 gD-nectin-1 complex [23]. It could be inferred that the above residues have significant interactions with the receptor, leading to a significant enhancement for binding. Inhibitors targeting to those residues may even block receptor nectin-1.

The ectodomain of nectin-1 consists of three Ig domains, an N-terminal variable-like domain (V domain) followed by two constant-like domains (C1 and C2 domain). The interaction pattern of BV gD-nectin-1 complex showed that the BV gD interaction sites were located at the V domain of nectin-1. Nectin-1 interacts gD mainly through five strands and some loops connecting them. The five strands belong to a β-sheet. Among the all, some residues, such as Asn77, Leu90, Glu125, etc., gave more interactions to BV gD molecule.

### The interaction of BV gD with the receptor of its natural host

BV is a natural pathogen of monkeys. It has been shown that BV can use simian nectin-1 for entry. To give a picture of how BV interacts with its natural host, we constructed a BV-gD simian nectin-1 complex model based on the human nectin-1 and HSV-gD complex.

**Figure 5:**
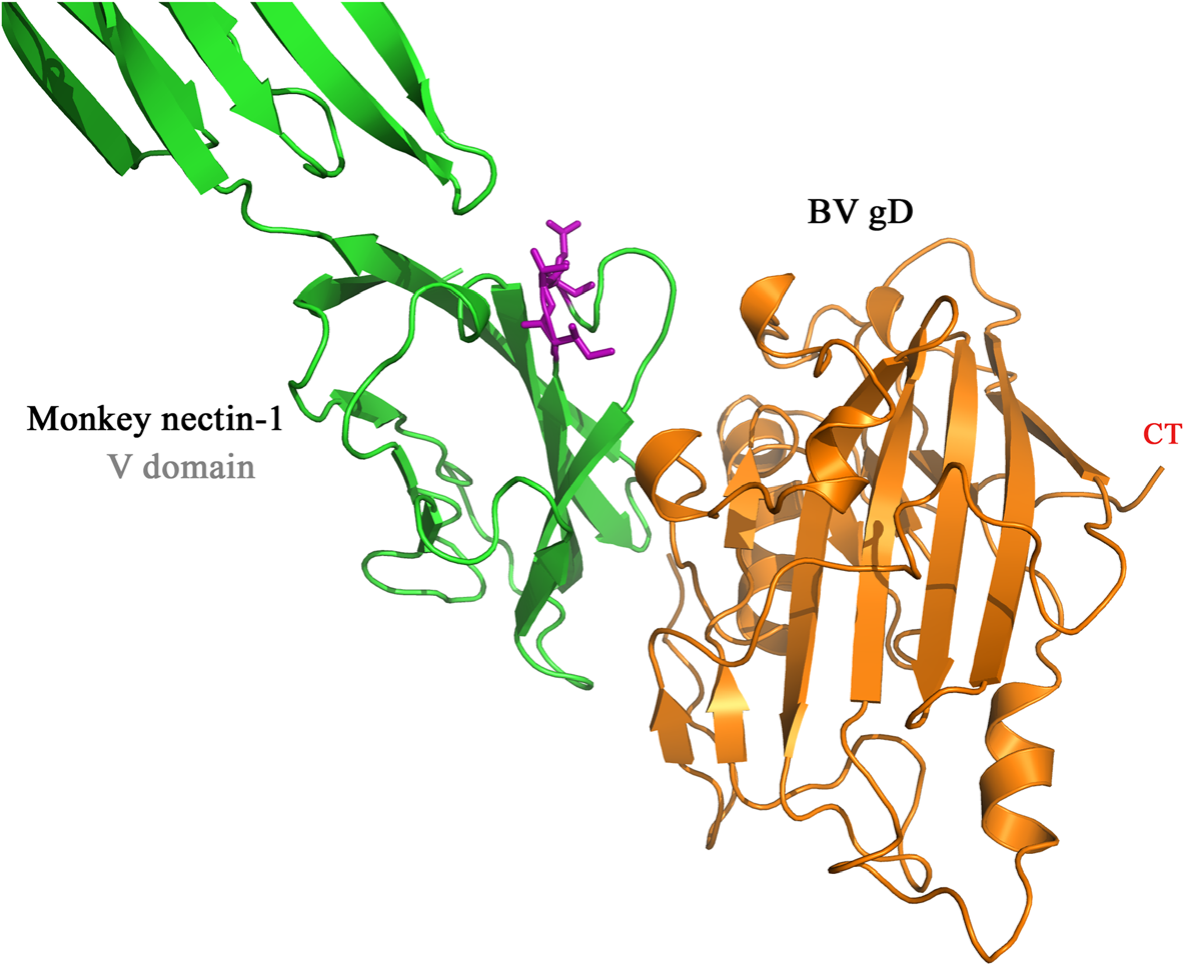
**The structure of BV gD-monkey nectin-1complex.** BV gD is colored orange, and monkey nectin-1 is colored green. The 3-amino acid insertion in monkey nectin-1 is highlighted in magenta and shown as sticks.

The pattern of BV-gD interaction with simian nectin-1(Figure 5) exhibited strong similarities to and slight differences with that of BV-gD with human nectin-1. The predicted BV-gD simian nectin-1 interface buries 1619 Å^2^ of solvent-accessible surface and has a shape complementarity value of 0.54, both values are almost identical to those of BV-gD human nectin-1.

There are 5 intermolecular hydrogen bonds and 52 hydrophobic interactions in the modelled BV-gD simian nectin-1 complex. The hydrogen bonds and hydrophobic interactions in this complex model are listed in Tables 4 and 5, respectively.

**Table 4:** Hydrogen bond interactions observed in the complex between BV gD- monkey nectin-1 complex

**Donor atom**	**Acceptor atom**	**Distance**	**Angle XDA**	**Angle DAY**
Nectin-1:Gln64:NE2	BV gD:Pro222:O	3.04	65.30	129.98
Nectin-1:Asn80:N	BV gD:Asp216:O	2.47	108.92	155.88
Nectin-1:Asn80:ND2	BV gD:Met220:O	3.08	158.26	109.72
BV gD:Tyr38:OH	Nectin-1:Gly89:O	3.07	118.93	129.15
BV gD:Arg223:NH2	Nectin-1:Glu128:OE2	2.91	120.46	134.88

**Table 5:** Hydrophobic interactions observed in the complex between BV gD- monkey nectin-1 complex

**BV gD residues**	**Monkey nectin-1 residues**
Pro24(2)	Pro130(2)
Arg36(1)	Met88(1)
Val37(2)	Met88(2)
Tyr38(14)	Ile83(1),Asn85(1), Met88(15)
Leu199(1)	Gly76(1)
Asp216(14)	Asn80(5),Leu93(9)
Gly217(2)	Gln79(2)
Gly219(2)	Asn80(2)
Leu221(3)	Thr66(3)
Pro222(1)	Gln64(1)
Phe224(4)	Thr63(4)
Ile231(1)	Phe132(1)
Val232(2)	Phe132(2)
Tyr235(4)	Pro133(4)

Nectin-1 is highly conserved among mammalian species. Monkey nectin-1 extracellular domain amino acid sequence is 98% identical to that of human nectin-1 with 2 amino acid substitutions and a 3-amino acid insertion after residue Lys-69 of the human nectin-1 V domain [32]. The 3-amino acid insertion in monkey nectin-1 falls within an extended region between two beta strands and is outside of the predicted gD-binding region.

The small changes in the intermolecular interactions of the two complexes are likely caused by the 3-amino acid insertion in monkey nectin-1 sequence. Although the 3-amino acid insertion does not directly interact with BV gD molecule, it is located in the loop region that connected two strands, which are constituting part of the ligand-binding region of nectin-1. Insertions at this loop might affect the interaction of the nearby strands, altering the shape of the nectin-1 interface. This alteration in binding surface would affect ligand binding. Similar phenomena had been observed in other protein complex [33,34].

### Why HVEM does not functions as an entry receptor for BV

**Figure 6:**
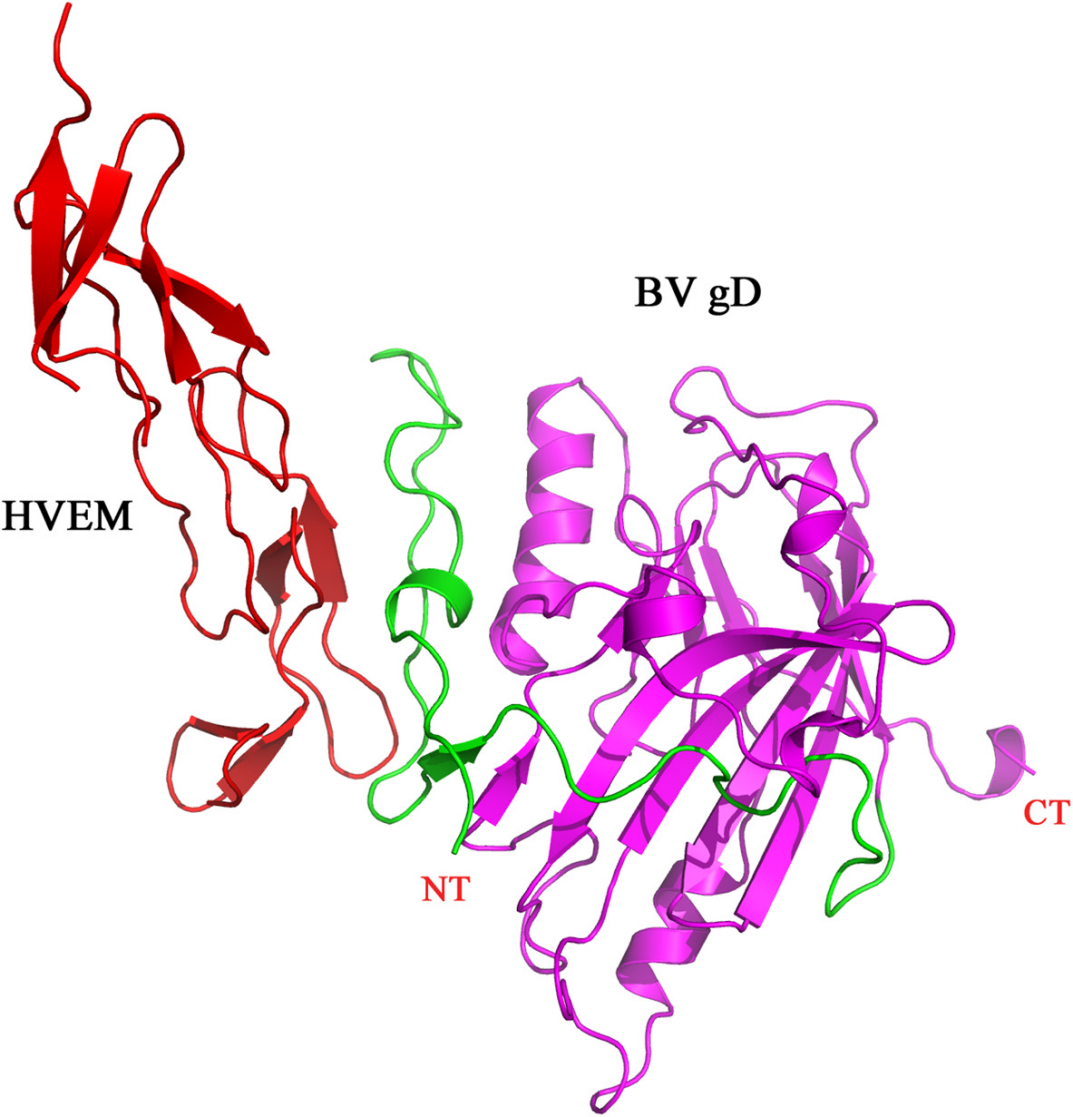
**The “hypothetical” BV gD-HVEM complex model.** The HVEM is colored red. The N-terminal extension of BV gD is colored green, the C-terminal extension and the central V domain are colored magenta.

A previous study reported that only nectin-1 is the primary receptor that mediates cell-cell fusion and virus entry for BV and HVEM does not function as entry receptor for BV [21,22]. To understand why HVEM does not mediate cell fusion by BV gD, we constructed a “hypothetical” BV gD-HVEM complex model and then compared the structure of this complex with that of HSV-1 gD-HVEM complex, and analyzed the changes in the intermolecular interactions of the two complexes.The overall structure of the constructed hypothetical BV gD-HVEM complex is shown in Figure 6. It has a very low shape complementarity factor of only SC = 0.32, which is markedly smaller than the value observed in the HSV-1 gD-HVEM complex (SC = 0.76).

The difference in shape complementarity correlates with buried surface decrease in the hypothetical BV gD-HVEM complex, which drops from 1432 Å^2^ for HSV-1 gD-HVEM to 877 Å^2^ for the hypothetical BV gD-HVEM complex. The two indices have previously been used to evaluate the extent of packing in interacting macromolecules [35]. The SC value measures the correlation between the shapes of the interacting surfaces, whereas the size of interface area reflects the extent of the contact between two molecules [36,37].

The small size of the buried surface and low SC value indicate that the interaction between BV gD and HVEM is weak. In the predicted BV gD-HVEM model, only a few intermolecular interactions were found, including 2 hydrogen bonds and 17 hydrophobic contacts (Table 6). The two figures are significantly smaller than those observed in HSV-1 gD-HVEM complex (Table 7). The lessening in intermolecular interactions greatly decreases the binding stability. It can be deduced that BV gD and HEVM be not able to form a stable complex because of the bad binding affinity between them.

**Table 6:** Intermolecular hydrogen bond interactions observed in the “hypothetical” BV gD-HVEM complex

**Donor atom**	**Acceptor atom**	**Distance**	**Angle XDA**	**Angle DAY**
BV gD:Arg11:NH1	HVEM:Cys37:O	2.78	83.98	95.04
BV gD:Arg11:NH2	HVEM:Cys37:O	2.84	81.55	70.25

**Table 7:** gD-HVEM interfaces

**Ligand**	**Receptor**	**Interface area (Å**^**2**^**)**	**SC**	**H-bonds**	**Hydrophobic contacts**
HSV-1 gD	HVEM	1431.357	0.757	10	25
BV gD	HVEM	877.726	0.316	2	17

Extensive mutagenic analysis of HSV-1 gD has been performed [38]. It has been found that amino acid substitutions on gD may abrogate physical and functional interactions of gD with HVEM. The inability of gD mutants to bind HVEM may have two causes [39]. First, the mutation may directly affect an interaction with HVEM. Second, the mutation may prevent proper formation of the hairpin.

It has been noted that Leu25 and Gln27 are critical for gD to form a the N terminal hairpin conformation [38]. Point mutations at position 25 and/or 27 are sufficient to abrogate HVEM binding, possibly because of poor formation of the hairpin [39,40]. Sequence analysis revealed that the BV gD and HSV-1 gD sequences share the same amino acid residues at position 25 and 27 (Figure 1). Based on the above observation, it can be inferred that BV-gD has the ability to form a N-terminal hairpin conformation. In fact, in our constructed BV gD-HVEM model, BV-gD N-terminus adopts a hairpin structure (Figure 6).

To gain insights into the sequence changes that contribute to the bad affinity of BV gD with HVEM, we undertook a comprehensive evaluation of the energetic contributions of all the residues in the HVEM binding interface of the HSV-1 gD. All positions that have different residues in BV-gD and HSV-1 gD within the HEAM binding interface were considered. According to sequence comparison, BV gD and HSV-1 gD differ at 15 positions within HVEM binding region. The binding free energies between a set of HSV-1 gD mutants and HVEM were estimated with the FoldX program. The HSV-1 gD mutants were focused on the 15 positions which have different residue with BV gD, and each position was mutated to the corresponding residue at the equivalent position of BV gD.

The absolute values of the free energies are meaningless since the FoldX forcefield is not scaled to fit the real free energy, but the relative difference between calculations is meaningful. The difference in the binding energies of the mutant and wild type structures can be used to estimate the effect of the amino acid variation on the stability of the protein-protein interaction. The predicted binding free energies of mutant HSV-1 gD-HVEM complexes were compared with the WT complex. The results are presented in Table 8. According to the calculation, the binding free energies for most substitutions compared with the WT complex were positive, these results implicate that most substitutions from HSV-1 gD residues to BV gD ones are likely to diminish the binding of HSV-1 gD with HVEM.

**Table 8:** Changes in binding free energies between HSV-1 gD - HEVM complex resulting from the replacement of HSV-1 gD residues with BV gD ones

**HSV-1 gD mutant**		**ΔΔ** ***G (kcal mol***^***−1***^***)***
**Position**	**Substitution**	
4	Leu → Pro	0.004
5	Ala → Val	−0.495
6	Asp → Glu	−0.044
7	Ala → Arg	−0.631
10	Lys → Thr	1.591
11	Met → Arg	1.751
12	Ala → Val	1.016
13	Asp → Asn	0.245
15	Asn → Gly	0.909
20	Lys → Ala	0.051
21	Asp → His	0.081
23	Pro → Ala	0.713
24	Val → Pro	0.204
26	Asp → Glu	−0.230
28	Leu → Lys	0.219
total		5.387

## Discussion

It is believed that the binding of BV gD to a cell surface receptor leads to membrane fusion and viral entry. Nectin-1 is known to be the “master” receptor for B virus infection. Up to now, the information about the sequence and structural requirements of the BV gD interaction with its fusion receptor is lacking because there is no structural data on either BV gD or the BV gD-nectin-1 complex available. In order to investigate how BV gD interacts with its receptor, the 3D structure of the BV gD ECD was constructed using homology modelling. Homology modelling, also referred to as comparative modelling, is currently the most accurate computational method for protein structure prediction. A limitation of homology modelling is that the quality of the predicted model strongly depends on the sequence identity between the target and the template.

The predicted BV gD-nectin-1 model showed that the interface between the BV gD and the receptor molecule was not geometrically complementary. The detailed analysis of the interactions between BV gD and nectin-1 suggested that hydrophobic contacts between these two molecules play key roles in their recognition and binding. Residues within the N- and C-terminal extensions of BV gD provided more interactions with nectin-1.

The constructed BV gD-HVEM complex model provided a structural basis for the reason why HVEM does not functions as a receptor for BV. In our observations, the BV gD-HVEM complex had a lower shape complementarity factor and a smaller interface area compared with the HSV-1 gD-HVEM complex and provided fewer intermolecular interactions. Binding energy calculations mutant HSV-1 gD-HVEM complexes showed that most mutants from HSV-1 gD residues to BV gD ones are likely to reduce the binding affinity of HSV-1 gD HVEM interaction. These results could explain why HVEM does not mediate BV fusion and entry process.

In this study, a modelling approach was successfully applied to generate the structures of BV gD and its receptors. The predicted complex model provides us with features that are consistent with the experimental data and can be considered a rough approximation of how BV gD might interact with its receptor. Finally, these results open new avenues for the computer-aided design of potential inhibitors or antagonists for blocking BV entry and infection.

## Conclusion

By applying molecular modelling and protein docking methods, three-dimensional structures of two BV gD-receptor complexes were built. With these structures, we obtained useful information about the detailed interaction between BV gD and its receptors. These models provide a glimpse of a receptor-ligand interaction that can lead to membrane fusion mediated by herpes B virus.

## Methods

### Data sets

Sequences of BV-gD and HSV-1 gD in FASTA format were retrieved from the public database UniProt/ExPASy (Swiss Bioinformatics Resource) http://expasy.org/tools/[41], having accession numbers of Q8UYF8 and Q05059 respectively. The sequence for Cercopithecus nectin-1 was retrieved from the GenBank data base with the accession number AF308635.

### Molecular modelling

Searches for reference proteins, sequence alignments and homology modelling were performed in Discovery Studio 2.5 (Accelrys Inc.). A BLAST search of the PDB indicated that the crystal structures of HSV-1 gD were suitable template models for the BV-gD. The full-length sequence identity between the BV-gD and the templates is relatively high (62%), the presence of high sequence conservation between the target and templates suggests that the comparative homology modelling of the BV-gD using the HSV-1 gD structure as template was appropriate.

To explore the molecular basis for the binding modes of BV-gD to receptor, its structure should be constructed in “receptor-bound form”. In this paper, structure of HSV-1 gD in complex with receptor were selected for the templates for homology modeling. The final templates selected for homology modeling were the HSV-1 gD crystal structure in nectin-1 binding form (PDB code 3U82) and HVEM binding form (PDB code 1JMA).

The quality of the predicted structure was evaluated using the Verify Protein (Profiles- 3D) protocol and Ramachandran Plot.

Profiles-3D Verify checks the validity of a protein structure by measuring the compatibility score of each residue in the given 3D environment. Scores for each residue and the whole protein are reported, and the expected high and low scores for a protein of the same size are given as a reference point [25].

The Ramachandran Plot indicates low energy conformations for φ and ψ, the conventional terms used to represent the torsion angles on either side of alpha carbons in peptides. This plot often serves as an important check to verify torsion angles in proteins. The Ramachandran Plot provides a graphical representation of the local backbone conformation of each residue in a protein. Each point on the Ramachandran Plot represents the φ and ψ torsion angles of a residue in protein. The plot also includes a representation of the allowed and disallowed regions for all residues. According to the plot, one can determine if individual residues are probably to be built correctly. The Ramachandran plot computed in here is as updated by Richardson and coworkers [26].

### Complex structure construction

Structural superposition is the fastest and easiest method for docking a ligand into an active site [42]. A number of studies have constructed complex models by the superposition approach [42-47]. Based on the fact that the gD of alphaherpesvirus subfamily binds to the same entry receptor in a similar fashion at a common site, we prepared the BV-gD-receptor model by structural superimposition using crystal structure of HSV-1 gD –nectin-1 complex as template. The putative binding site in BV gD and the known receptor binding site in HSV-1 gD were superimposed. The BV-gD-receptor structure was obtained by superposition of the structure of BV-gD onto the structure of HSV-1 gD in complex with nectin-1, and after removal of the template HSV-1 gD.

### Protein–protein interaction analysis

The interface area of a complex is derived from its atomic coordinates by computing its accessible surface area in solvent and subtracting it from the sum of the accessible surface areas of the isolated components [48]. Accessible surface areas were evaluated with the program SURVOL [49], which implements the Lee and Richards algorithm [50]. Group radii were from ref [51], and the radius of the water probe was 1.4 Å.

The shape complementarity (SC) value is used to check how well two adjacent surfaces fit together by taking into account distances and angles between both surfaces [52]. The SC value was calculated by the program SC.exe, which is part of the CCP4 software package (Collaborative Computational Project Number 4 1994).

A hydrophobic contact is defined as a distance between carbon atoms shorter than 4.0 Å [53]. All intermolecular hydrophobic contacts are listed.

Protein structure illustrations were generated with the PyMOL Molecular Graphics Software [54].

### Interaction free energy estimation

The binding affinity of a complex is strongly correlated to its relative binding free energy. To compare the relative binding affinities of the gD-receptor complexes, we calculated the relative binding free energies for these complexes using the FoldX program [55-57]. FoldX uses a full atomic description of the structure of the proteins. The different energy terms taken into account in FoldX have been weighted using empirical data obtained from protein engineering experiments. The FoldX energy function includes terms that have been found to be important for protein stability.

## Abbreviations

BV: B virus; gD: glycoprotein D; SC: Shape complementarity; HSV: Herpes simplex virus; HVEM: Herpesvirus entry mediator.

## Competing interests

The authors declare that they have no competing interests.

## Authors’ contributions

JY, YL1 and WG formulated the study. LL and ZQ performed the research. YL4 and FL analysed the data. HY and YC participated in the analysis and discussion. JY and LL wrote the manuscript. All authors read and approved the final manuscript.
